# Auditory Contagious Yawning Is Highest Between Friends and Family Members: Support to the Emotional Bias Hypothesis

**DOI:** 10.3389/fpsyg.2020.00442

**Published:** 2020-04-03

**Authors:** Ivan Norscia, Anna Zanoli, Marco Gamba, Elisabetta Palagi

**Affiliations:** ^1^Department of Life Sciences and Systems Biology, School of Nature Sciences, University of Turin, Turin, Italy; ^2^Unit of Ethology, Department of Biology, University of Pisa, Pisa, Italy

**Keywords:** emotional contagion, bottom-up attention, selective attention, top-down attention, yawn contagion, mimicry

## Abstract

Contagious yawning differs from spontaneous yawning because it occurs when an individual yawns in response to someone else’s yawn. In *Homo sapiens* and some non-human primates contagious yawning is higher between strongly than weakly bonded individuals. Up to date, it is still unclear whether this social asymmetry underlies emotional contagion (a basic form of empathy preferentially involving familiar individuals) as predicted by the Emotional Bias Hypothesis (EBH) or is linked to a top-down, selective visual attention bias (with selective attention being preferentially directed toward familiar faces) as predicted by the Attentional Bias Hypothesis (ABH). To verify whether the visual attentional bias explained the yawn contagion bias or not, in this study, we considered only yawns that could be heard but not seen by potential responders (auditory yawns). Around 294 of auditory yawning occurrences were extrapolated from over 2000 yawning bouts collected in free ranging humans for over nine years. Via GLMM, we tested the effect of intrinsic features (i.e., gender and age) and social bond (from strangers to family members) on yawn. The individual identity of the subjects (trigger and potential responder) was included as random factor. The social bond significantly predicted the occurrence of auditory yawn contagion, which was highest between friends and family members. A gender bias was also observed, with women responding most frequently to others’ yawns and men being responded to most frequently by others. These results confirm that social bond is per se one of the main drivers of the differences in yawn contagion rates between individuals in support of the EBH of yawn contagion.

## Introduction

Yawning is an involuntary sequence of mouth opening, deep inspiration, brief apnea, and more or less slow expiration (Baenninger, 1997; Walusinski and Deputte, 2004; Guggisberg et al., 2010; Krestel et al., 2018). When elicited, a yawn cannot be totally suppressed. Therefore, it has been defined as a stereotyped or reflex-like pattern (Lehmann, 1979; Provine, 1986). In *Homo sapiens*, several hypotheses have been put forth with variable support to explain mechanisms and functions of spontaneous yawning, such as oxygenation (respiratory function caused by hypoxia), stress-related behavior (caused by arousal), or thermoregulation (caused by hyperthermia; Guggisberg et al., 2010; Massen et al., 2014; Gallup and Gallup, 2019). Being a physiological response, yawning can be affected by internal and external factors such as the time of the day (Giganti and Zilli, 2011) or intracranial/brain temperature (Gallup and Eldakar, 2013).

Yawning can be self-directed and/or displayed to others (Moyaho et al., 2017; Palagi et al., 2019). In human and non-human primates, depending on the species, when yawning is shown to others, it can communicate threat (Troisi et al., 1990; Deputte, 1994) and/or physiological and behavioral changes (Provine et al., 1987; Leone et al., 2015; Zannella et al., 2015). In humans, yawning is a socially modulated response because it can be inhibited by actual—and not virtual—social presence (Gallup et al., 2019) and because a yawn can be triggered by someone else’s yawn, as a result of a phenomenon known as contagious yawning (Provine, 1989, 2005). Yawn contagion can be elicited even if the yawn is heard but not seen (Arnott et al., 2009; Massen et al., 2015).

In humans, their phylogenetically closest ape species (chimpanzees: *Pan paniscus*; bonobos: *Pan troglodytes*) and the African monkey *Theropithecus gelada*, contagious yawning is not only present (Provine, 1986; Palagi et al., 2009; Tan et al., 2017; but see: Amici et al., 2014) but also socially modulated because the yawning response is highest when certain categories of individuals are involved (e.g., kin, group-members, dominants; Palagi et al., 2009; Campbell and de Waal, 2011, 2014; Norscia and Palagi, 2011; Demuru and Palagi, 2012; Massen et al., 2012). Two main arguments have been presented to explain this social asymmetry in contagious yawning, which have been grouped into two main hypotheses: the *Emotional Bias Hypothesis* (EBH), linking contagious yawning to emotional transfer, and the *Attentional Bias Hypothesis* (ABH), which considers contagious yawning as a motor response that is subject to differences in top-down attentional processes (Palagi et al., 2020).

The EBH predicts that the social asymmetry observed in yawn contagion rates reflects differences in the different social bonding, a proxy of emotional bonding, between individuals. This hypothesis is supported by evidence that yawn contagion rates follow an empathic trend (*sensu* Preston and de Waal, 2002), being highest between individuals sharing a strongest emotional bond. Specifically, Norscia and Palagi (2011) found that in humans yawn contagion rates are greatest in response to kin and friends than in response to acquaintances and strangers. In adult chimpanzees yawn contagion is higher between in-group compared to out-group members (Campbell and de Waal, 2011) and in bonobos yawn contagion rates are greatest between individuals that affiliate more with one another (Demuru and Palagi, 2012). In a comparative investigation including both humans and bonobos, Palagi et al. (2014) found that the yawn contagion rates were affected by the relationship quality between individuals more than by the species the subjects belonged to. Additionally, in humans yawn contagion increases with age when the ability to identify others’ emotions increases and declines with old age when such ability declines (Wiggers and van Lieshout, 1985; Anderson and Meno, 2003; Saxe et al., 2004; Singer, 2006; Millen and Anderson, 2011; Bartholomew and Cirulli, 2014). Yawn contagion rates increase from infancy to adulthood also in chimpanzees (Madsen and Persson, 2013).

The ABH predicts that the social asymmetry observed in yawn contagion can be due to differences in social, visual attention (Massen and Gallup, 2017). In particular, highest levels of contagious yawning would be due to the extra top-down, selective visual attention paid to individuals that are more relevant to the observer, such as familiar subjects, as it occurs in humans and geladas, or dominants, as it occurs in chimpanzees or bonobos (Yoon and Tennie, 2010; Massen et al., 2012; Massen and Gallup, 2017). According to Massen and Gallup (2017), ABH would be backed up by the existing evidence on the different visual detection and visual perceptive encoding of faces of familiar and/or in-group subjects compared to unfamiliar ones (e.g., Buttle and Raymond, 2003; Ganel and Goshen-Gottstein, 2004; Jackson and Raymond, 2006; Michel et al., 2006).

In this study, we analyzed data on yawning collected over 9 years on humans in their natural settings and we extrapolated the cases in which the yawn emitted by a subject could be heard but not seen by a potential responder (auditory yawn). By considering only the cases in which the visual cue of the yawning stimulus was not detectable, we verified whether the social asymmetry previously observed in yawn contagion rates persisted or not. In particular, we tested the following alternative predictions derived from the two hypotheses presented above (EBH and ABH).

Prediction 1a: according to the EBH, the rates of yawn contagion are influenced by the strength of the inter-individual social bond—a proxy of the emotional bond—*per se* and not by a different top-down, selective visual attention paid to certain individuals in particular. If this hypothesis is supported, we expect to observe the social bias also when the visual cue of the yawning stimulus is excluded and the rates of auditory contagious yawning to be higher between strongly bonded compared to weakly bonded individuals.

Prediction 1b: according to the ABH, the higher levels of yawn contagion between strongly bonded compared to weakly bonded individuals would be linked to the closest top-down, selective visual attention that individuals pay to individuals that are relevant to them, e.g., family and friends. If this hypothesis is supported, the social bias observed in the yawn contagion should disappear when only auditory yawns are considered because the visual cue cannot be attended by the potential responder.

## Materials and Methods

### Data Collection and Operational Definitions

For this study, we considered the vocalized yawns emitted by a subject that could only be heard—but not seen—by a potential responder (hereafter: auditory yawns). The emitter and the potential responders had to be in a range of ≤5 m. Vocalized yawns involved the use of vocal folds and the yawns that only involved heavy inspiration/expiration were not considered as vocalized; 294 cases of auditory yawns were extrapolated from a dataset of a total of 2001 yawning bouts collected over 9 years—from 2010 to 2019—by using the all occurrences sampling method (Altmann, 1974). Specifically, auditory yawns were collected from November 2010 to May 2019, from 05.30 am to 02.30 am, on human Caucasian subjects, aged from 18 to 77, during their routinely activities, e.g., in work places, over meals, during social meetings, etc., with the subjects being unaware of being observed and in absence of any evident external source of anxiety. The auditory yawn database included 193 yawner-potential responder dyads. Depending on the situation, the information was recorded, unnoted, through alphanumerical codes and entered directly into calculation sheets, typed in mobile phones or written on paper, and then entered in calculation sheets for subsequent elaboration. Basic information such as age and the relationship between people was known to the authors. The potential responders were coded as in the non-sight condition when their head was rotated by 180° with respect to the trigger or when a physical, sight-blocking obstacle was present preventing the potential responder from seeing the trigger’s face and body. Trigger and responder were never completely isolated (e.g., in two separate rooms with closed doors) from one another. The social closeness was collected on four levels: 0 = strangers, who had never met before; 1 = acquaintances, who exclusively shared an indirect relationship based on a third external element, that is work duty (colleagues) or friends in common (friends of friends); 2 = friends, non-related individuals sharing a direct relationship not exclusively related to a third external element; 3 = regular partners and kin (*r* ≥ 0.25). Previous literature reports that yawn responses can be elicited within 5 min after watching someone else’s yawn (the trigger’s yawn) (Provine, 1986), with a maximum in the first minute (Provine, 2005; Palagi et al., 2014). Literature also reports that from the fourth minute there is a highest probability of autocorrelation (meaning that the presence of a yawn performed by a subject at t_0_ increases the probability to have another yawn by the same subject at t_(__0+X)_ where X is the increasing unit of time; Kapitány and Nielsen, 2017). Hence, we considered the yawn responses occurring within a 3 min time window from the yawn emitted by the trigger. To further reduce the autocorrelation bias, in case of a chain of yawns emitted by the trigger (more yawns emitted in the 3-min time window) we registered as a response only the first yawn performed after the perception of the last yawn. We coded a yawn as “spontaneous” when no other subject had yawned in the 5 min preceding the yawning event.

### Statistical Analyses

For the analyses, the following variables were considered: occurrence of contagion, coded as: 1 = presence, 0 = absence; the social bond was entered with the four levels defined above (0 = strangers; 1 = acquaintances; 2 = friends; 3 = kin); trigger’s and observer’s sex were labeled as: M = male, F = female; the age classes of the trigger and the responder were coded as follow: yo = youth (18–24 years old); ad = adult (25–64 years old); se = senior (above 65 years old) (Statistics Canada, 2009); the time slots were coded as follows: 1 = 05:30–09:00 am; 2 = 09:01 am–12:30 pm; 3 = 12:31–16:00 pm; 4 = 16:01–19:30 pm; 5 = 19:31–23:00 pm; 6 = 23:01–02:30 (Giganti and Zilli, 2011). The database (see Supplementary Data Sheet) included 84 males, 69 females, 16 youngsters (yo), 122 adults (ad), and 15 senior (se). To test whether the occurrence of yawn contagion was influenced by the factors bond (0 = strangers; 1 = acquaintances; 2 = friends; 3 = kin), sex of the trigger (Trigger_sex), sex of the responder (Responder_sex), the age class of the trigger (Trigger_ageclass), and the age class of the responder (Responder_ageclass), and time slot (from 1 to 6), we used a generalized linear mixed model (GLMM) that included these five predictors as fixed effects and triggers (Trigger) and responders (Responder) identities as random effects. We fitted the models in R (R Core Team, 2018; version 3.5.1) using the function lmer of the R-package lme4 (Bates et al., 2015). We established the significance of the full model by comparison to a null model comprising only the random effects (Forstmeier and Schielzeth, 2011). We used a likelihood ratio test (Dobson, 2002) to test this significance (ANOVA with argument “Chisq”). We calculated the *p*-values for the individual predictors based on likelihood ratio tests between the full and the null model by using the R-function “drop1” (Barr et al., 2013). As the response variable was binary, we used a binomial error distribution. We tested whether the interaction between the sexes or the age classes of the trigger and the responder were significant, but as they were not, we did not include them in the model. We used a multiple contrast package (multcomp) to perform all pairwise comparisons for each bonding levels with the Tukey test (Bretz et al., 2010). We reported the Bonferroni-adjusted *p*-values, estimate (Est), standard error (SE), and *z*-values.

## Results

**FIGURE 1:**
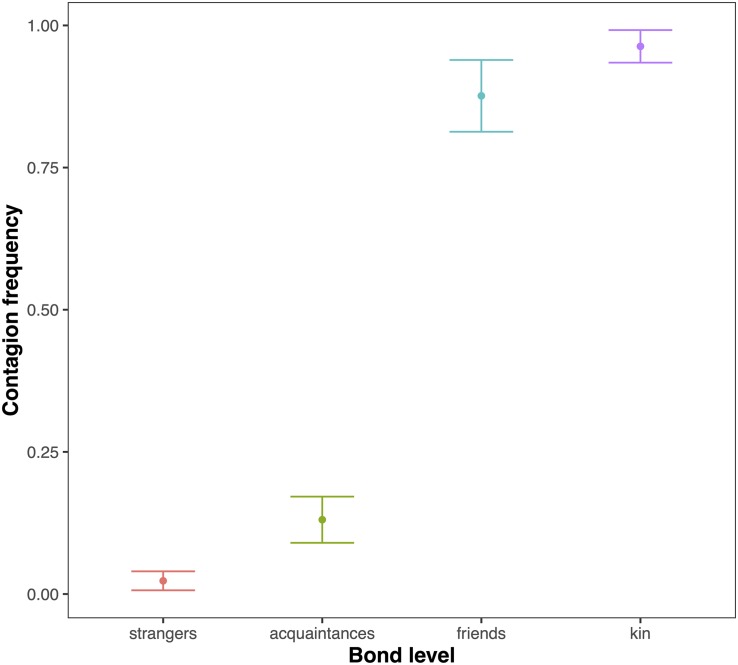
Line plot of the effect of the social bond between trigger and responder (*X*-axis) on the mean occurrence of acoustic yawn contagion (*Y*-axis). Friends and kin show significantly higher yawn contagion frequencies than strangers and acquaintances (Tukey test: friends versus strangers *p* < 0.001; kin versus strangers *p* < 0.001; friends versus acquaintances *p* < 0.001; kin versus acquaintances *p* < 0.001; other combinations, ns).

**FIGURE 2:**
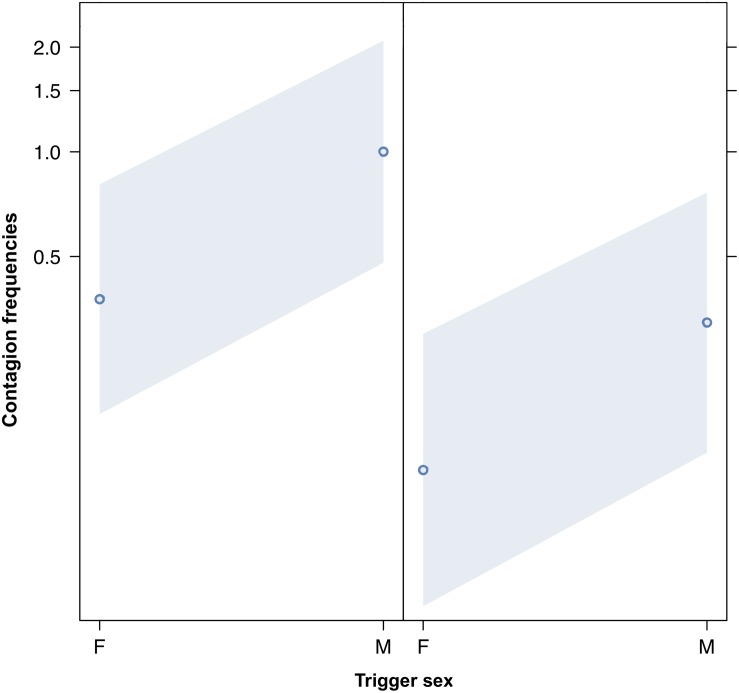
Line plot of the effect of the sex of the trigger (*X*-axis) on acoustic yawn contagion mean occurrence (*Y*-axis). Effect of the trigger sex on acoustic yawn contagion when the responder is a female (**right**) and a male (**left**). Males’ yawns elicit more yawns than females’ ones regardless of the sex of the responder (result of the GLMM, *p* = 0.022). Points represent the effect that the response variable has on the independent variable “sex of the trigger,” based on the value predicted by the model. Colored bands show the 95% confidence interval.

**FIGURE 3:**
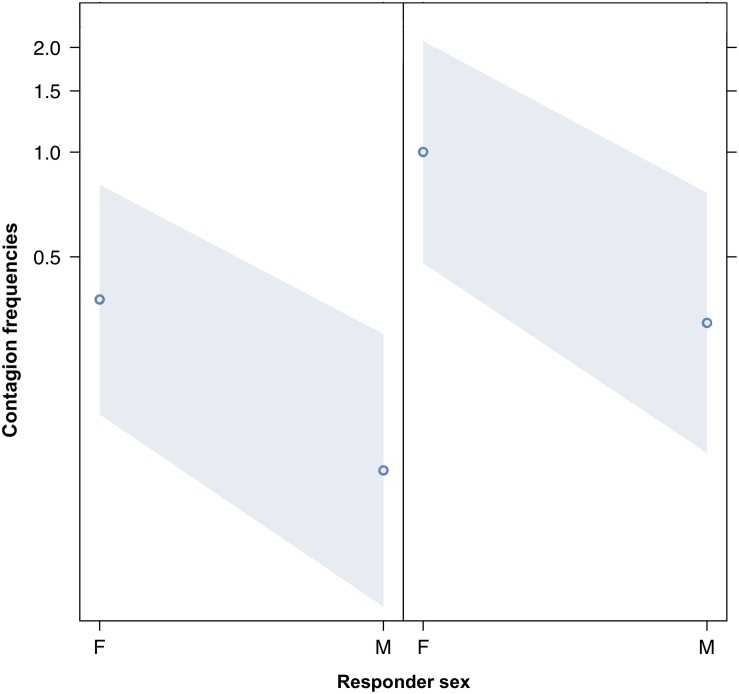
Line plot of the effect of the sex of the responder (*X*-axis) on acoustic yawn contagion mean occurrence (*Y*-axis). Effect of the responder sex on acoustic yawn contagion when the trigger is a female (**right**) or a male (**left**). Females respond significantly more than males regardless of the sex of the trigger (result of the GLMM, *p* = 0.021). Points represent the effect that the response variable has on the independent variable “sex of the responder,” based on the value predicted by the model. Colored bands show the 95% confidence interval.

We compared the model fitted versus a null model comprising only the random factors (likelihood ratio test: χ^2^ = 149.995, *df* = 17, *p* < 0.001). As we found at least one predictor was having a significant impact on the response, we moved on with a drop1 procedure. The GLMM indicated a significant effect of bond across four comparisons (Tukey test; 2 = friends versus 0 = strangers, Est = 5.4810; SE = 0.9807, *z* = 5.589, *p* < 0.001; 3 = kin versus 0 = strangers, Est = 6.6872, SE = 1.1602, *z* = 5.764, *p* < 0.001; 2 = friends versus 1 = acquaintances, Est = 3.7643, SE = 0.7201, *z* = 5.227, *p* < 0.001; kin = 3 versus 1 = acquaintances, Est = 4.9706, SE = 0.9381, *z* = 5.299, *p* < 0.001) (Figure 1). The pairwise comparisons of bonding levels revealed that yawn contagion was significantly higher in family and friends than in strangers and acquaintances, with no significant differences between strangers and acquaintances and between family and friends (1 = acquaintances versus 0 = strangers; Est = 1.7167, SE = 0.7062, *z* = 2.431, *p* = 0.0664; 3 = family versus 2 = friends; Est = 1.2063, SE = 0.8960, *z* = 1.346, *p* = 0.5156). The GLMM also indicated a significant effect of the sex of both triggers and responders, and of bonding (see Table 1): yawn contagion of female responders was higher compared to males (Figure 3), and males, as triggers, were responded to more frequently by others compared to females (Figure 2). In contrast, we found no significant main effects of the age of both triggers and responders, the time slot in which yawns were emitted, and interaction between the sex of the subjects (Table 1).

**TABLE 1:** Results of the GLMM, including the following fixed factors: bond (0 = strangers; 1 = acquaintances; 2 = friends; 3 = kin), trigger and responder sex (M = male; F = female), trigger and responder age class (yo = youth, 15–24 years old; ad = adult, 25–64 years old; se = senior, above 65 years old), and time slot (1 = 05:30–09:00 am; 2 = 09:01 am–12:30 pm; 3 = 12:31–16:00 pm; 4 = 16:01–19:30 pm; 5 = 19:31–23:00 pm; 6 = 23:01 pm–02:30 am).

	Estimate	SE	χ^2^	*P*
(Intercept)^a^	–2.404	0.956	a	a
Bond (acquaintances)^b,c^	1.844	0.722	2.554	0.000
Trigger sex (male)^b,c^	0.919	0.451	2.036	0.037
Responder sex (male)^b,c^	–1.207	0.512	–2.358	0.012
Trigger age class (senior)^b,c^	0.255	0.949	0.268	0.930
Responder age class (senior)^b,c^	–0.934	1.045	–0.893	0.722
Time slot (09:01–12:30)^b,c^	–1.011	0.929	–1.089	0.326

*The identity of triggers (Trigger) and responders (Responder) was included as random factors. Full versus null model: chisq = 149.995, df = 17, p < 0.001. ^a^Not shown as not having a meaningful interpretation. ^b^Estimate ± SE refer to the difference of the response between the reported level of this categorical predictor and the reference category of the same predictor. ^c^These predictors were dummy coded, with the “Bond (Strangers),” “Trigger sex (Female),” “Responder sex (Female),” “Trigger age class (Adult),” “Responder age class (Adult),” and “Time slot (05:30–09:00)” being the reference categories.*

## Discussion

This study shows for the first time that yawn contagion is significantly affected by the social bond between individuals (Table 1) even when the triggering stimuli are auditory yawns, which we defined as vocalized yawns that could be heard but not seen (visual cue undetectable, auditory cue detectable). In particular, auditory contagious yawning is significantly more frequent between kin and friends than between strangers and acquaintances (Figure 1). This finding supports prediction 1a based on the EBH and not prediction 1b based on the ABH, leading to the conclusion that in humans top-down, selective visual attention cannot be the main driver of the social asymmetry observed in yawn contagion rates (Norscia and Palagi, 2011; Norscia et al., 2016a). Also the sex of the trigger and the sex of the receiver had a significant effect on yawn contagion rates, with men—as triggers—being responded to by others more frequently than women (Figure 2) and women responding more frequently to others’ yawns than men (Figure 3).

Contrary to Bartholomew and Cirulli (2014), we found no age effect on yawn contagion, most probably because our database on auditory yawns had a strong prevalence of adults (25–64 years old). The highest levels of auditory yawn contagion in women compared to men confirm the gender bias observed in naturalistic conditions on humans susceptible to yawn contagion by Norscia et al. (2016a, b) when considering a larger dataset that also included yawns that could be seen by the potential responder (with yawning sensory modality—vision, hearing, or both—not affecting the response). The gender bias is also in partial agreement with previous results obtained in controlled settings, including the visual cue (Chan and Tseng, 2017; but see Norscia and Palagi, 2011; Bartholomew and Cirulli, 2014). It has been hypothesized that the high degree of yawn contagion in women might inform emotional contagion (Norscia et al., 2016a), in the light of reportedly higher empathic abilities—related to maternity—of women compared to men (Christov-Moore et al., 2014). However, this issue is still under debate because cultural differences across human societies can mold social bonding dynamics in a different way. It is therefore complicate, at this stage of knowledge, to disentangle cultural factors, inter-personal relationship quality, and gender influence in the distribution of yawn contagion. Our results also show that men perform better than women as triggers and the most parsimonious hypothesis for this might be that men’s vocalizations can be better heard in natural settings, often characterized by background noises. Indeed, the perception of voice gender primarily relies on the fundamental frequency that is on average lower by an octave in male than female voices, with lower frequency vocalizations traveling further than high frequency ones (Marten and Marler, 1977; Latinus and Taylor, 2012). However, to our knowledge, there is no specific study addressing the possible gender bias in yawn audibility and further investigation with experimental trials in controlled condition is therefore necessary to verify this speculation.

In this study, we also found that the differences in yawn contagion rates across categories (family and friends, strangers, and acquaintances) cannot be explained by differences in top-down, selective visual attention. This finding is in line with previous literature. Contagious yawning appears to involve brain areas that are more related to the orienting-bottom up network [temporoparietal junction (TPJ), brainstem nuclei, ventrolateral prefrontal cortex (vlPC)] than top-down related areas [frontal eye fields (FEFs), intraparietal sulcus (IPS), parietal areas; for a review: Palagi et al., 2020]. Moreover, yawn contagion is neither sensitive to the sensory cues present in the signal (auditory, visual, or audio-visual) (Arnott et al., 2009; Norscia and Palagi, 2011) nor affected by the visual perspective of the triggering stimulus (yawns in orientations of 90°, 180°, and 270° are able to trigger yawning responses as frontal, 0° yawns; Provine, 1989, 1996). Chan and Tseng (2017) found that the ability to detect a yawn as such (perceptual detection sensitivity) was related to the duration of gaze to the eyes of the stimulus releasing face, but eye-gaze patterns were not able modulate contagious yawning. In chimpanzees, contagious yawning frequencies were highest between same-group than different-group individuals, even if the responders looked longer at out-group chimpanzee videos (Campbell and de Waal, 2011). The argument that visual selective attention can bias yawn contagion rates in a specific direction (subjects responding more to family than strangers) is also undermined by the absence of any specific pattern of social attention in human and non-human primate. Via eye-tracking (applied to measure the viewing time) and by showing unknown faces to their experimental subjects, Méary et al. (2014) observed that humans were skewed toward own-race faces whereas rhesus macaques’ attention was more attracted by new than by same species faces. Kawakami et al. (2014) observed that human subjects paid more attention to the eyes of ethnic in-group members and to nose and mouth of ethnic out-group members. The same study also revealed that visual attention did not depend on the target race. By measuring how long the experimental subjects gazed at the screen, Whitehouse et al. (2016) observed that Barbary macaques paid more attention to scratching videos of non-stranger than stranger individuals but also noted that within the non-strangers, macaques paid most attention to those individuals with which they shared a weak social bonding. By measuring glance rates, Schino and Sciarretta (2016) observed that mandrills looked more at their own kin than at non-kin but also more at dominant than at subordinate group mates. Therefore, these studies (used to support ABH) describe no single pattern of selective attention. One further important point to consider is the very definition of familiarity and group-membership adopted by most of the studies used to support ABH (Massen and Gallup, 2017). These studies showed better visual detection and visual perceptive encoding of faces of familiar/in-group subjects compared to unfamiliar ones but defined familiarity and group membership *not* on the basis of the personal relationships between individuals. Instead, familiarity or group-membership were defined on the ground of indirect knowledge (e.g., photo of famous people or of a subject already shown in pre-trial phases) or common race (e.g., Buttle and Raymond, 2003; Ganel and Goshen-Gottstein, 2004; Jackson and Raymond, 2006; Michel et al., 2006). This definition is fine for the purposes of these studies but it is not as much fine if the results are used to propose alternative explanations for the influence that real social bonding—based on real relationships—may have on a phenomenon, in this case yawn contagion. For example, Michel et al. (2006) observed that Caucasian and Asian subjects could better recognize same-race faces but this difference was not present in Asian subjects who had been living for about a year among Caucasians. Another point of discussion concerns the presence of yawn contagion in children with autism spectrum disorder (ASD), which frequently show alterations in visual attention (Richard and Lajiness-O’Neill, 2015). In ASD children, yawn contagion can be absent (Senju et al., 2007), impaired (Helt et al., 2010), or similar to typically developing children when the subjects are induced to redirect their attention the video stimulus during the experimental trials (Usui et al., 2013). In a recent study, Mariscal et al. (2019) found that yawn contagion in ASD children was positively related to the blood concentration of oxytocin, the hormone involved in parental and social attachment (Decety et al., 2016) and posited that yawn contagion in ASD children may be related to variable mean oxytocin concentrations across different study cohorts (Mariscal et al., 2019). This finding is in line with the EBH hypothesis that links yawn contagion rates to social bonding, which can reflect emotional bonding.

## Conclusion

Our study adds to the discussion over the mechanisms underlying the social asymmetry in yawn contagion (for a critical reviews: see Adriaense et al., 2020; Palagi et al., 2020), by showing that yawn contagion is probably associated with bottom-up, rather than with top-down, selective attention. Bottom-up attention is primarily lead by the sensory perception of the eliciting stimulus whereas top-down, selective attention is a voluntary, sustained process in which a particular item is selected internally and focused upon or examined (Katsuki and Constantinidis, 2014). In this respect, the acoustic stimulus (auditory yawn) emitted by the trigger was heard and could elicit a yawning response in the receiver, even though the receiver was not paying any voluntary visual attention to the trigger. Moreover, the yawning response rates were socially modulated, with auditory yawn contagion being highest in individuals that were most strongly bonded to one another. Hence, top-down selective attention is not the main driver of the social asymmetry observed in yawn contagion, which appears to be a stimulus driven phenomenon-related to bottom-up attention processes. Further investigation is necessary to understand whether and in what way other forms of attention or pre-attentive stages are able to affect yawn contagion.

## Data Availability Statement

The dataset used for this study is provided in the Supplementary Material.

## Ethics Statement

The study involving human participants were reviewed and approved by Comitato di Bioetica d’Ateneo (University Bioethical Committee)—University of Turin (ref. no. 451945). Written informed consent for participation was not required for this study in accordance with the national legislation and the institutional requirements.

## Author Contributions

IN and EP carried out data collection and conceived and wrote the manuscript. AZ helped with data collection and manuscript revision, figures, and tables. MG carried out statistical analyses and wrote the related part of the manuscript.

## Conflict of Interest

The authors declare that the research was conducted in the absence of any commercial or financial relationships that could be construed as a potential conflict of interest.
